# Test-Retest Reliability of Two Computationally-Characterised Affective Bias Tasks

**DOI:** 10.5334/cpsy.92

**Published:** 2024-12-18

**Authors:** Alexandra C. Pike, Katrina H. T. Tan, Hoda Tromblee, Michelle Wing, Oliver J. Robinson

**Affiliations:** 1 Department of Psychology, University of York, UK; 2 Anxiety Lab, Neuroscience and Mental Health Group, Institute of Cognitive Neuroscience, University College London, UK; 3 Department of Clinical, Educational and Health Psychology, University College London, UK

**Keywords:** affective bias, anxiety, depression, test-retest reliability, measurement, psychometrics

## Abstract

Affective biases are commonly seen in disorders such as depression and anxiety, where individuals may show attention towards and preferential processing of negative or threatening stimuli. Affective biases have been shown to change with effective intervention: randomized controlled trials into these biases and the mechanisms that underpin them may allow greater understanding of how interventions can be improved and their success be maximized. For such trials to be informative, we must have reliable ways of measuring affective bias over time, so we can detect how and whether they are altered by interventions: the test-retest reliability of our measures puts an upper bound on our ability to detect any changes. In this online study we therefore examined the test-retest reliability of two behavioural affective bias tasks (an ‘Ambiguous Midpoint’ and a ‘Go-Nogo’ task). 58 individuals recruited from the general population completed the tasks twice, with at least 14 days in between sessions. We analysed the reliability of both summary statistics and parameters from computational models using Pearson’s correlations and intra-class correlations. Standard summary statistic measures from these affective bias tasks had reliabilities ranging from 0.18 (poor) to 0.49 (moderate). Parameters from computational modelling of these tasks were in many cases less reliable than summary statistics. However, embedding the covariance between sessions within the generative modelling framework resulted in higher estimates of stability. We conclude that measures from these affective bias tasks are moderately reliable, but further work to improve the reliability of these tasks would improve still further our ability to draw inferences in randomized trials.

## Introduction

Affective bias may be defined as the tendency to preferentially process or respond to a specific class (e.g. negative or positive) of emotionally-relevant stimuli or tasks. Affective bias is commonly studied within mental health research, as *negative* affective biases are frequently observed in disorders such as depression and anxiety (Beck, 1979; MacLeod et al., 1986; MacLeod & Mathews, 2012). These biases may occur in memory (Ellwart et al., 2003; Herrera et al., 2017; Marchetti et al., 2018), attention (Bar-Haim et al., 2007; Peckham et al., 2010; Robinson et al., 2013), or in the interpretation of ambiguity (Aylward et al., 2020; Everaert et al., 2017), are thought to be present in risk groups (Chan et al., 2007; van Oostrom et al., 2013), may predict the onset of depression (Forbes et al., 2007; Smith et al., 2018). Therefore, negative affective bias seems to play an important role in affective disorders (Roiser et al., 2012).

Currently, we have limited understanding of how many of the treatments for anxiety and depression work. However, a single dose of an antidepressant has been shown to attenuate affective biases, potentially providing a mechanism by which antidepressants have their effect (Harmer et al., 2009). Randomized controlled studies that examine and contrast the effects of different treatments on affective biases may provide mechanistic insights into what different treatments have in common and how they differ, and extend this early work in antidepressants.

In order to perform these randomized controlled studies, we need reliable and valid ways of measuring affective bias over time in the same individuals. In particular, if estimates of affective bias are not stable over time within individuals then any effect of treatment is likely to be obscured or confounded. In other words, the ‘test-retest’ reliability of affective bias measures puts an upper limit on our ability to detect the impact of interventions.

Two common tasks which measure forms of affective bias are the ‘ambiguous midpoint’ task (Aylward et al., 2020) and the ‘go-nogo task’ (Guitart-Masip et al., 2012; Mkrtchian et al., 2017). The ‘ambiguous midpoint’ task elicits a measure of biased decision-making: participants are shown two stimuli (circles, or tones), and learn to associate each with different responses (button presses), which lead to outcome magnitudes (two different sizes of reward). After these associations have been learnt, participants are shown a stimulus which is at the midpoint of the two they have learnt, and have to respond using a button press. The tendency to produce the response associated with the smaller magnitude reward (or larger magnitude punishment) given the presentation of the midpoint stimulus is a form of pessimistic bias: this is elevated in those with anxiety and depression (Aylward et al., 2020; Daniel-Watanabe et al., 2020).

During the ‘go-nogo task’, participants are presented with four stimuli in turn, each of which they must learn the appropriate response to. They can either choose to respond (‘go’) or not respond (‘no-go’), and they will either obtain rewards or punishments. Unbeknownst to them, each of the four stimuli corresponds to a different combination of outcome and response: they should respond to obtain a win in the ‘go to win’ condition, not respond to obtain a win in the ‘no-go to win’ condition, respond to avoid a loss in the ‘go to avoid’ condition, and not respond to avoid a loss in the ‘no-go to avoid’ condition. A learner who was performing purely in accordance with the goals of the task would be able to learn all of these stimuli equally, and show equivalent accuracy in responding to each of them throughout the task. However, participants tend to exhibit a ‘Pavlovian bias’, where they are more successful at associating ‘go’ responses with ‘win’ outcomes, and ‘no-go’ responses with ‘avoid’ outcomes, which is known as Pavlovian-instrumental transfer (Huys et al., 2011). In particular, avoidance can be operationally defined as more accurate responding to ‘no-go to avoid’ rather than ‘go to avoid’ stimuli, or the tendency to withhold action in the face of (potential) negative outcomes. When this task is modelled, participants with anxiety or depression may show an enhanced ‘avoid bias’, or tendency to associate ‘no-go’ responses with ‘avoid’ outcomes, alongside generally lower accuracy (Mkrtchian et al., 2017).

Unlike many other affective bias tasks, both of these tasks have been extensively characterized using computational modelling, which, importantly may improve their test-retest reliability (Haines et al., 2020; Hedge et al., 2020; Price et al., 2019; Shahar et al., 2019). This improvement in reliability may emerge from the use of *generative* models, which allow researchers to estimate underlying parameters that are assumed to capture a latent process that generates behaviour. These latent processes are thus often assumed to be more generalizable than taking summary statistics over task performance, and more reliable (Haines et al., 2020; Hedge et al., 2020), though these assumptions are rarely interrogated (Brown et al., 2020). Of course, this may depend on the task: those with good test-retest reliability for model-free measures also tend to show good parameter recovery (Smith et al., 2018). Furthermore, the model-fitting approach employed may also have large effects (Brown et al., 2020), as may over-parameterization of models. Finally, if the task does not capture different levels of the cognitive process of interest (inter-individual variability), computational modelling will not be more reliable than summary statistics (Hedge et al., 2020). As a result, a wide range of test-retest reliability values have been reported for computational models in the field of affective bias research: ranging from poor (Moutoussis et al., 2018; Weidinger et al., 2019) to good and excellent (Brown et al., 2020; Chung et al., 2017; Mkrtchian et al., 2023; Smith et al., 2018).

In this study, we therefore aimed to examine the test-retest reliability of participants’ performance on two illustrative computationally-characterised affective bias tasks, the ‘ambiguous midpoint’ and ‘go nogo’ affective bias tasks, using both summary statistic and computational modelling measures.

## Methods and Materials

### Ethical Approval

This study was approved by the UCL Research Ethics Committee (15253/001). Prior to starting the tasks, participants were provided with an information sheet and consent form to complete online.

### Participants

Participants were recruited online using Prolific.ac (Palan & Schitter, 2018), and all study tasks and questionnaires were presented using Gorilla (Anwyl-Irvine et al., 2020). Note that the use of Prolific allows for participant anonymization: Prolific assigns participants identifier codes, and we have no access to their names, email addresses, or contact details. Prior to making data available online, we stripped all variables that were not essential for analysis, including Prolific IDs, and used our own randomly assigned participant numbers instead. See the supplement for sample size justification.

All participants were required to be aged 18–60, fluent in English, and with no history of mild cognitive impairment or dementia.

Participants were reimbursed at a rate of £7.50 per hour (and were not paid any ‘bonus’ sums, regardless of their rewards obtained in the tasks), and a minimum of two weeks passed between the first and second testing sessions.

### Procedure

Participants read an online information sheet, gave informed consent, and filled out a questionnaire on demographic and mental health variables (see Supplementary Material). Participants then completed two mental health questionnaires: the Personal Health Questionnaire (8 item version, excluding the suicide item; PHQ-8) (Kroenke et al., 2001, 2009), and the Generalized Anxiety Disorder scale (GAD-7) (Spitzer et al., 2006, p. 7).

Finally, participants completed the go-nogo task and the ambiguous midpoint task (see ‘Tasks’ below), and were redirected to Prolific.

Participants completed the second session two weeks later, following the same steps, but omitting the PHQ-8 and GAD-7. This two-week interval was chosen to reflect a common duration between sessions in randomized controlled trials and clinical studies, whilst more broadly balancing the need for stability of relevant factors (e.g. mood) with effects of fatigue or task memory from close-together testing sessions.

### Tasks

#### go-nogo task

**Figure 1:**
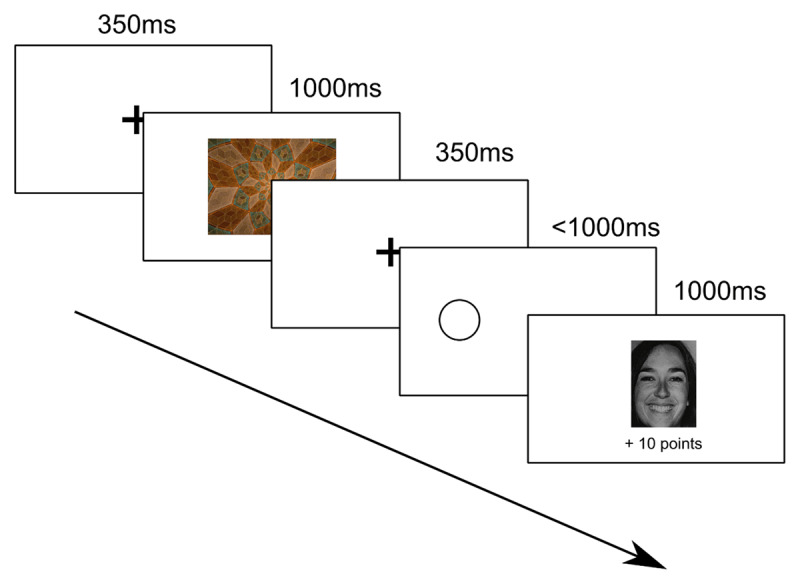
Schematic of go-nogo task. After the presentation of a fixation cross (250 ms), participants were shown a fractal image (1000 ms). After a brief pause (100ms) a fixation cross was presented (250ms). This was followed by another pause (100ms), after which participants were shown a fractal image (1000ms). After another pause (100ms) fixation cross (250ms) and another pause (100ms) a circle was presented. After another fixation cross (250 ms), a circle was presented, on either the left or the right side of the screen. On each trial, participants could either respond (Go) or not respond (No-go) to the presentation of the circle. If they chose to respond, this response consisted of pressing the ‘a’ keyboard key if the circle was on the left of the screen, or the ‘l’ keyboard key of the circle was on the right of the screen. Note that these keys were not randomised, as the response keys (‘a’ for the left side, and ‘l’ for the right side) was designed to be consistent with a standard UK/US keyboard layout. This screen automatically timed-out after 1000 ms, even if the participant had not responded. Subsequently, feedback was shown on the screen for 1000 ms. A ‘reward’ consisted of the presentation of a happy face and text saying ‘+10 points’, a ‘neutral’ outcome was the presentation of a horizontal yellow bar with the text ‘0 points’, and a ‘punishment’ outcome was an unhappy face with the text ‘–10 points’.

During this task (Figure 1) participants had to learn the correct response to make in order to obtain rewards and avoid punishments. There were four trial types, each corresponding to one fractal stimulus, and participants had to learn whether the correct choice was to respond (‘go’) or withhold a response (‘nogo’) to each of the four stimuli. There were 160 trials in total (with no training phase), all of which were randomised (i.e. an interleaved design), with each fractal appearing 40 times. Stimuli were either rewarding, and accompanied by a reward or neutral feedback; or punishing, and accompanied by either neutral feedback or a punishment. Outcomes were probabilistic: 80% of the time, the ‘correct’ response to a stimulus (go or nogo) was followed by the associated feedback; 20% of the time, the feedback was misleading. Faces could be either male or female, and were shown in a random order.

#### Ambiguous midpoint task

**Figure 2:**
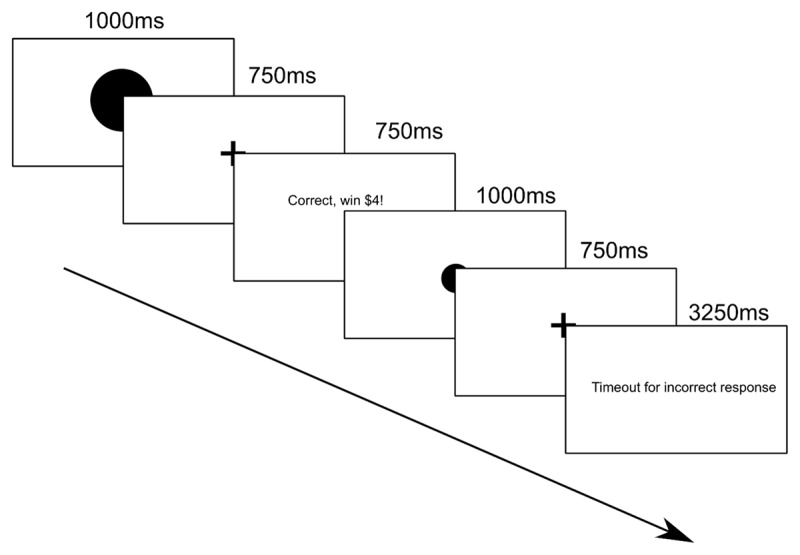
Schematic of ambiguous midpoint task. Participants were shown a circle for 1000 ms, followed by a fixation cross for 750 ms. Participants responded using the ‘m’ and ‘z’ keyboard keys. There were three sizes of circles – small and large – which were each associated fully deterministically with one key and one size of reward – and one medium sized circle which was associated 50% with the ‘m’ key and corresponding reward, and 50% with the ‘z’ key and corresponding reward. If participants responded correctly, they were shown the message ‘Correct, win $4’ or ‘Correct, win $1’ depending on whether the stimulus presented was associated with a larger or smaller reward. This feedback was presented for 750 ms. If participants responded incorrectly, they were shown the message ‘Timeout for incorrect response’, which lasted for 3250 ms. If they failed to respond in time, they were shown the message ‘Too late, timeout!’ which also lasted for 3250 ms.

This task (Figure 2) was divided into an initial ‘training’ phase, and then the main task. In the training phase, which consisted of 20 trials, participants were presented with two circles: one large, and one small. Each of these circles was associated with a particular key press – either ‘z’ or ‘m’, and a different size of reward – either $1 or $4. During the training phase, participants learnt which of the two keys they should press in response to which size of circle, and which size of reward they could expect as a result. The associations were fully deterministic – they were always rewarded with the same size of reward if they pressed the correct key.

During the main task, which consisted of 120 trials, participants were presented with these circle stimuli (40 trials of each) or a circle which was exactly in between the sizes of the two circles they had learnt to respond to during the training phase (also presented for 40 trials). These trials were shown in the same randomized order to each participant. They were told to respond using the key corresponding to the circle closest in size: in reality, 50% of the time the ‘z’ key was ‘correct’ (i.e. led to a reward) and 50% of the time the ‘m’ key was correct. Note that participants would receive the corresponding-sized rewards if they responded using the key that happened to be ‘correct’ on that trial. The response buttons were not randomized in this version of the task, to ensure that all participants experienced the same key-to-response mapping in both sessions.

As the midpoint stimulus was ambiguous – i.e. was not actually closer in size to the smaller or larger circle – the extent to which participants responded by pressing the key associated with the circle that delivered a smaller reward is a measure of negative affective bias.

### Analysis

Data were analysed in R, version 4.1.0. All code and data is openly available on the OSF and Github, at: https://doi.org/10.17605/OSF.IO/PUK9E.

We calculated the test-retest reliability of the tasks in several ways: see below.

#### Indices

##### Summary statistics

We calculated summary statistics that were appropriate to each task, and which are generally used as indices of ‘negative affective bias’.

Summary statistics for the go-nogo task consisted of the mean accuracy of response to each of the four stimulus types. More specifically, we report the proportion of trials in which participants responded using the correct key on ‘go to win’ (respond to gain a reward) and ‘go to avoid’ (respond to avoid a punishment) trials, and the proportion of trials for which participants *withheld* a response on ‘no-go to win’ (do not respond to get a reward) and ‘no-go to avoid’ (do not respond to avoid a punishment) trials.

For the ambiguous midpoint task, we used the mean proportion of medium sized circles to which participants responded by pressing the key associated with the larger reward. This is referred to throughout as ‘p(high|mid)’ (or the probability of a response associated with a high reward given a medium stimulus) – which refers to the proportion of medium sized circles to which a participant responded with the keypress associated with higher reward. Note that a larger value of p(high|mid) represents less negative affective bias – this could be reported as 1-p(high|mid) or p(low|mid) for a more intuitive readout of ‘negative affective bias’.

##### Model parameters

We also used generative models to calculate parameters reflecting participants’ learning and decision-making for each task.

For the go-nogo task, we used a set of models from the hBayesDM package (Ahn et al., 2017). We also examined extended versions of these models based on previous work (Mkrtchian et al., 2017). These models capture how participants learn about the different stimuli based on outcomes received (a reinforcement learning framework), including parameters that quantify approach-avoidance Pavlovian bias and a bias towards making ‘go’ responses. More details can be found in the Supplement.

We used drift-diffusion models to capture performance on the ambiguous-midpoint task (Aylward et al., 2020). We fit both an approximation to the drift-diffusion model (Wagenmakers et al., 2007), and a 4-parameter drift-diffusion model instantiated in hBayesDM (Ahn et al., 2017). These models capture the gradual accumulation of evidence before a choice is made when evidence reaches a decision boundary, with parameters that govern the starting point of the accumulation process, how far apart the decision boundaries are, and the rate at which evidence accumulates (drift rate). More details can be found in the Supplement.

#### Reliability calculations

##### Correlation analysis

We calculated Pearson’s correlations between these metrics at time 1 and time 2.

##### Intra-class correlation coefficients

We also calculated intra-class correlation coefficients (ICC). We used both absolute agreement and the equivalent consistency ICCs: absolute agreement ICCs are sensitive to mean differences between timepoints, and consistency ICCs reflect overall rank order. We used two-way ICCs, with a fixed effect of time, and a random effect of participant. These are equivalent to ICC(A,1) and ICC (C,1) (McGraw & Wong, 1996), and the consistency ICC is also known as an ICC(3,1) (Shrout & Fleiss, 1979). ICCs of zero indicate low or no reliability, with 1 indicating perfect reliability. Typically, ICCs below 0.4 indicates poor reliability, 0.4 to 0.75 indicates moderate-good reliability, and above 0.75 suggests excellent reliability (Fleiss, 1999).

##### Posterior predictive performance

If model parameters are reliable, then parameters from session 1 should be better able to predict performance on session 2 than chance (Mkrtchian et al., 2023). Additionally, though, because the partial pooling used by specifying higher-level priors in hierarchical Bayesian estimation can result in ‘shrinkage’ – i.e. parameter estimates tend to be pulled closer to the group mean – it is also important to check whether *group mean* parameters from session 1 are better able to predict an individual’s performance on session 2 than that *individuals’ own* parameters are.

To address the first of these points, we calculated the mean of the likelihood of the choices participants actually made on each trial of session 2, given their best-fitting parameters from session 1 (and vice versa). We also repeated this using the mean parameters in each session, to test whether any effects were just due to shrinkage. Note that we did not correct for multiple comparisons here, as this analysis is designed to complement the main tests performed in this paper and provide additional information about shrinkage.

##### Correlation matrix embedded within generative model

Rather than fitting generative models to participant data from each session separately, it is also possible to fit the models to each session together and incorporate the correlation matrix between sessions into the models (see Supplement) (Haines et al., 2020). This has significant benefits: compared to extracting parameter means from fitted models and correlating them, estimating the correlation within the model itself allows the uncertainty around the parameter values to be incorporated, and also allows Bayesian priors to be set over possible values of the correlation matrix, thus resulting in fully Bayesian inference. As more of the uncertainty is accounted for (specifically, imprecision in the estimation method), higher confidence can be held in the correlation estimates.

Given the nature of this approach, it is only theoretically meaningful to do this type of estimation with generative models (in which we believe that the parameters reflect an underlying process which is consistent over time), rather than summary statistics.

## Results

### Participants

84 participants completed session 1, and 64 completed session 2. Participants were excluded if they had an accuracy of 0 on ‘go to win’ trials in either session (indicating that they weren’t responding at all to this stimulus – three participants, one in session 1, two in session 2), or made no responses at all in either task (two participants, one of whom also had an accuracy of 0 on ‘go to win’ trials in session 2), or used only one response key in the ambiguous midpoint task (0 participants). Additionally, one participant never responded with the button corresponding to the ‘high reward’ circle for the medium circle size presentation in the Ambiguous Midpoint task, so their data could not be modelled and they were excluded. One further participant refreshed the task so their data could not be modelled (they had duplicate trials), so they were also excluded. After data exclusions, we had 58 participants.

Participants had a mean age of 28.00 (sd = 8.43), and a mean Prolific score (see Supplementary Material for definition) of 99.43/100 (sd = 1.67). Participants scored a mean of 7.86 (5.29) on the PHQ-8, and a mean of 6.60 (4.88) on the GAD-7. 34 of the 58 participants were female, 23 were male, and one was nonbinary. All participants had a gap between session 1 and session 2 of at least 14 days (mean = 15.67, sd = 3.60, minimum = 14, maximum = 32). More details about participants’ characteristics, including mental health variables, can be found in the Supplementary Materials.

### Summary Statistics

**Figure 3:**
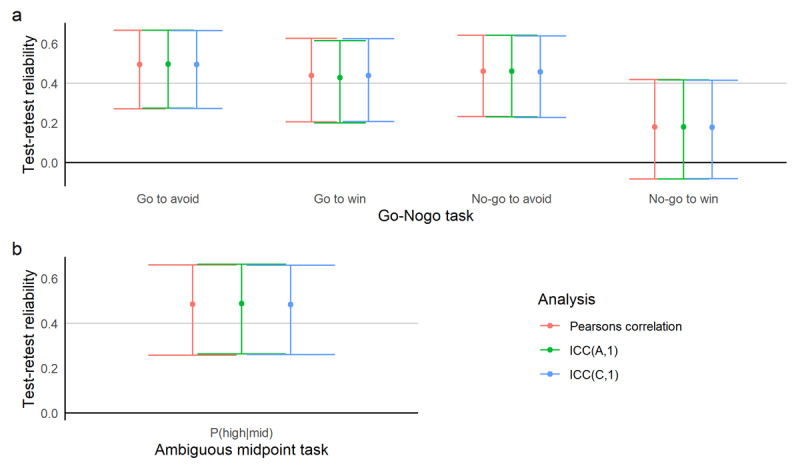
Pearson’s correlation coefficients and Intra-Class Correlation Coefficients for summary statistic measures from each affective bias task: **a)** Go-nogo task, **b)** Ambiguous midpoint task. The X axis shows the relevant summary statistics from the specific task, y axis shows the estimate and the 95% confidence interval around them. The black line at y = 0 represents a correlation or ICC of 0 – confidence intervals that do not cross this represent significant estimates. Points above the light grey line at y = 0.4 represent estimates that show reliability of moderate or above; points below this line indicate poor reliability.

Correlations and ICCs between all commonly-used summary statistics were significant, and all were within the moderate-good range (Figure 3, Table 1), with the exception of ‘no-go to win’ accuracy on the go-nogo task, which was poor. Individual performance per condition of each participant can be seen in the Supplementary Materials.

**Table 1:** Pearson’s correlation coefficients and Intra-Class Correlation Coefficients for summary statistic measures from each affective bias task. Values greater than 0.4 are often described as moderate-good, less than 0.4 are poor.


TASK	MEASURE	PEARSON’S CORRELATION		ICC(A,1)		ICC(C,1)	
		
		COEFFICIENT	p-VALUE	COEFFICIENT	p-VALUE	COEFFICIENT	p-VALUE

**go-nogo**	Go to win accuracy	0.439	0.0006	0.430	0.0002	0.440	0.0002

	Go to avoid accuracy	0.495	<0.0001	0.496	<0.0001	0.495	<0.0001

	No-go to win accuracy	0.179	0.1786	0.180	0.0886	0.178	0.0888

	No-go to avoid accuracy	0.462	0.0003	0.461	0.0001	0.457	0.0001

**Ambiguous midpoint**	P (high|mid)	0.484	0.0001	0.488	<0.0001	0.484	<0.0001


### Model Parameters

#### go-nogo task

Model comparison indicated that the best-fitting model was one with a learning rate, bias towards making rather than withholding responses (go bias), separate Pavlovian approach and avoid biases, reward and punishment sensitivity, and a parameter governing how deterministically participants responded (see Supplement). This model is similar to the best-fitting model in our previous work using this task, except in the number of learning rates (Mkrtchian et al., 2017).

**Figure 4:**
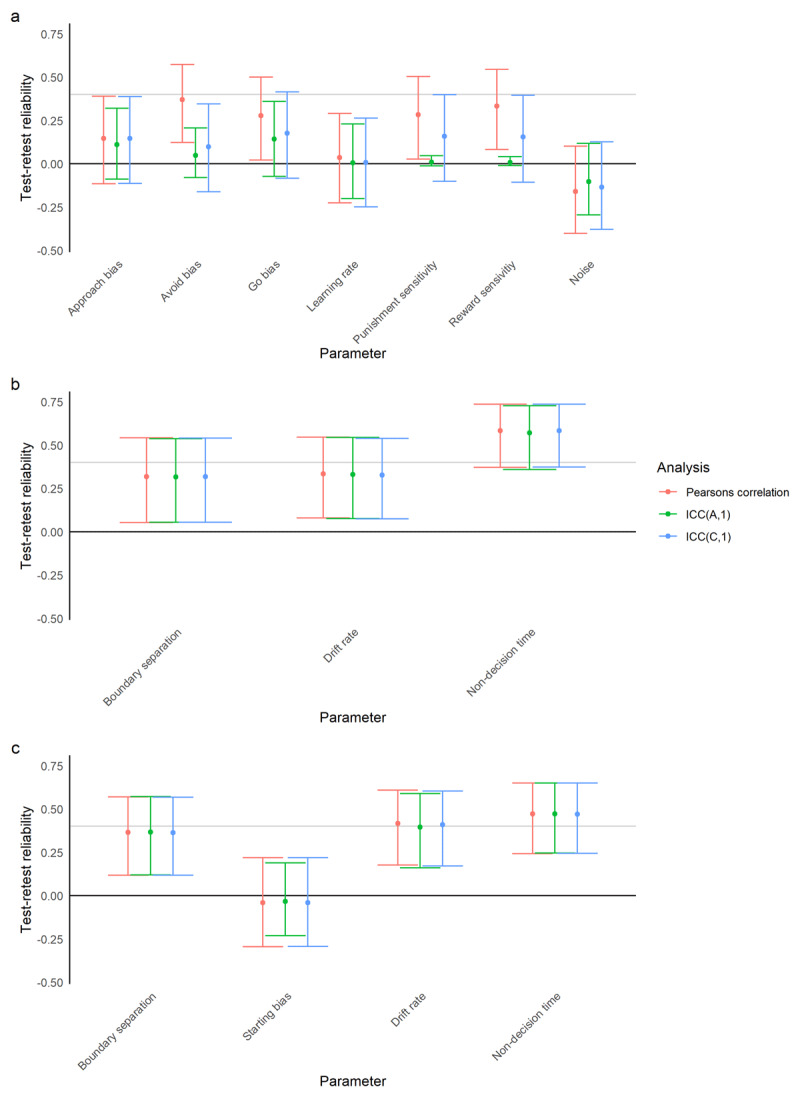
Pearson’s correlation coefficients and Intra-Class Correlation Coefficients for parameters from different models fit to the affective bias tasks. X axis shows parameter, y axis shows the estimate and the 95% confidence interval around it for the three different measures of test-retest reliability used. The black line at y = 0 represents a correlation or ICC of 0 – confidence intervals that do not cross this represent significant estimates. Points above the grey line at y = 0.4 represent estimates that show reliability of moderate or above; points below this line indicate poor reliability. **A)** The model that best fit choice data on the go-nogo task, **B)** a simplified drift diffusion model fit to the ambiguous midpoint task, **C)** the 4-parameter drift diffusion model fit to the ambiguous midpoint task.

The correlations between parameters at times 1 and 2 were variable. These are displayed in Figure 4A. In particular, no parameter showed a statistically different ICC estimate from 0, although the Pearson’s correlations between several parameters at time 1 and time 2 (avoid bias, go bias, reward and punishment sensitivity) were significant.

#### Ambiguous midpoint task

We fit both an approximation of the Drift Diffusion Model (EZDDM; Wagenmakers et al., 2007), (Figure 4B), which omits the starting bias term and has a closed-form solution, and a 4-parameter Drift Diffusion Model (Figure 4C). Notably, the test-retest reliability for the parameters included in both models was similar.

### Posterior Predictive Performance

#### go-nogo task

**Figure 5:**
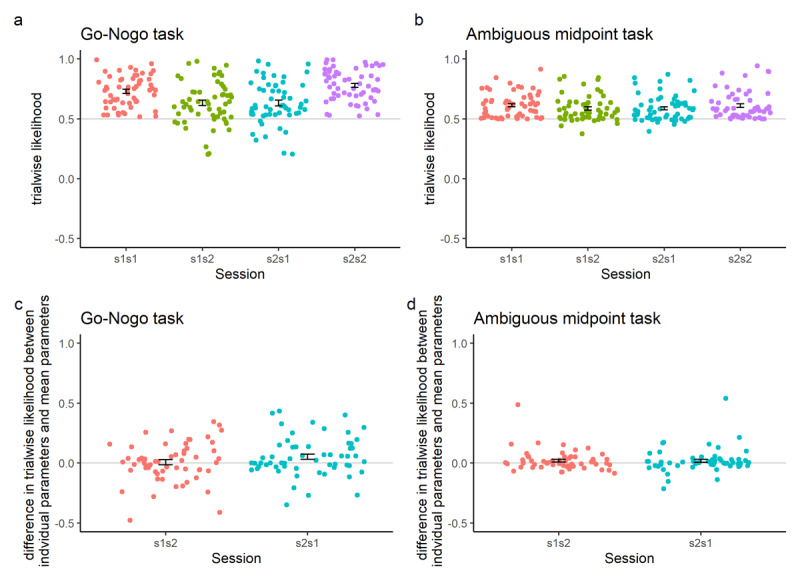
Posterior predictive performance of model parameters, displayed using scatterplots (one individual is represented by each point) and error bars showing the standard error around the mean. Each individual’s model parameters from different sessions were used to calculate the trialwise likelihood of their actual choices made during that session for the go-nogo task **(a)** and the ambiguous midpoint task **(b)**. To check whether better-than-chance prediction was due to shrinkage resulting from the partial pooling used in hierarchical Bayesian model fitting, the mean parameters from each session were used to predict each individual participant’s choices in the go-nogo task **(c)** and the ambiguous midpoint task **(d)**. The first number in all x axis names refers to the session from which the parameters were drawn; the second refers to the session on which performance was predicted, thus ‘s2s1’ refers to the use of parameters from session 2 to predict session 1 performance. The grey lines represent the values against which t-tests were performed (i.e. chance).

For the go-nogo task, each individual’s parameters from session 1 were able to predict performance on session 2 better than chance (t-test against 0.5, *t*_57_ = 5.74, *p* < 0.001; Figure 5A), as were parameters from session 2 able to predict performance on session 1 (*t*_57_ = 5.70, *p* < 0.001). However, there was no significant difference between using each individual’s session 1 parameters or the mean session 1 parameters to predict session 2 trialwise likelihood (t-test of difference against 0, *t*_57_ = 0.278, *p* = 0.782; Figure 5C), but each individual’s parameters session 2 parameters were significantly better at predicting session 1 performance than the mean session 2 parameters (*t*_57_ = 2.45, *p* = 0.017).

#### Ambiguous midpoint task

Each individual’s parameters from session 1 were better able to predict performance on session 2 than chance (*t*_57_ = 6.30, *p* < 0.001; Figure 5B). Parameters from session 2 were also better able to predict performance on session 1 than chance (*t*_57_ = 6.35, *p* < 0.001). Individual’s parameters did not out-perform mean parameters in predicting performance for either session (session 1 parameters prediction session 2: *t*_57_ = 1.80, *p* = 0.076; session 2 parameters predicting session 1: *t*_57_ = 1.40, *p* = 0.166; Figure 5D).

### Embedded correlation matrix

**Figure 6:**
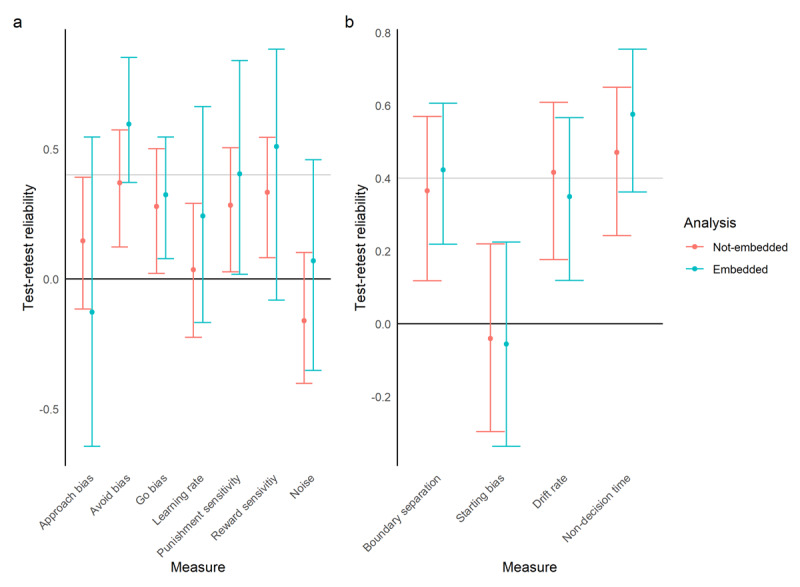
Pearson’s correlation coefficients and estimates of the correlations when these are embedded within a generative model, for parameters from different models fit to the affective bias tasks. X axis shows parameter, y axis shows the estimate of each measure of test-retest reliability and the 95% confidence interval around it. The black line at y = 0 represents a correlation of 0 – confidence intervals that do not cross this represent significant estimates. Points above the grey line at y = 0.4 represent estimates that show reliability of moderate or above; points below this line indicate poor reliability. **A)** The model that best fit choice data on the go-nogo task, **B)** the 4-parameter drift diffusion model fit to data on the ambiguous midpoint task.

When we estimated the correlations between parameters at time 1 and time 2 using the same generative model, results were somewhat similar (Figure 6). Notably, the estimates were higher when correlation matrices were embedded, particularly for the go-nogo task and the drift rate parameter in the drift diffusion model. The exceptions are approach bias on the go-nogo task (which was substantially lower, albeit with greater variance), and the starting bias and drift rate on the ambiguous midpoint task (which were slightly lower).

## Discussion

The test-retest reliability of the two affective bias tasks measured in this study ranges from low to good. Summary statistic measures had ICCs which were all greater than 0.4 (except nogo-to-win, which had an ICC of 0.17). Computational parameters derived from generative models of these tasks generally had worse reliability.

Summary statistics measured on these tasks had generally moderate to good test-retest reliability. Whilst there is some cause for optimism in that all but one of these estimates were greater than 0.4 and statistically significantly different to 0, thus indicating that there is some reliable latent factor being measured robustly over time, the magnitude of these relationships has implications for study power. In particular, when these measures are used in clinical research (such as clinical trials or randomized controlled studies of mechanisms), their moderate reliability will set an upper bound on any observed effect. Imagine, for example, that an intermediate endpoint in a clinical trial was the proportion of medium sized circles to which participants responded with the keypress associated with higher reward. A far greater sample size would be necessary to see a statistically significant change in this measure compared to baseline for a measure with lower reliability, as there will be greater variance in the difference between participants’ scores over time. To be specific, if the anticipated effect size of an SSRI was 0.55 (Otto et al., 2001), 17 participants would be needed to detect a change in a paired-samples two-tailed t-test if the measure had a test-retest reliability of 0.48; if this was 0.9, the same effect could be seen in only 11 participants. Using a more conservative recent estimate of 0.27 for the effect of an SSRI, the corresponding number of participants needed would be 59 (0.48 test-retest reliability) or 15 (0.9 test-retest reliability).

We had hoped that using computational modelling might improve matters (Brown et al., 2020; Haines et al., 2020; Price et al., 2019; Shahar et al., 2019), given that the premise of this modelling is the assumption that generative models capture latent factors which are causally responsible for generating the (noisy) behaviour that we observe in participants. As our results show, these computational parameters were not more reliable than summary statistics. This could be driven by issues with the assumptions underlying computational modelling: perhaps these parameters do not represent real latent factors and are thus not more ‘valid’ than the summary statistics conventionally used; perhaps these latent factors exist but are not stable, and change depending on the context (as is increasingly recognized to be the case with learning rate, which adjusts depending on task volatility; (Behrens et al., 2007; Pulcu & Browning, 2017); or perhaps the model-fitting procedure (or the specific models used in this paper) are not adequately capturing these latent variables. It is likely, examining Figures 4 and 6, that the correct answer is some combination: it seems that some model parameters are more reliable than others, perhaps capturing real constructs, and that others are not, perhaps just acting to ‘mop up’ residual noise in participant choices. However, the parameters that have differed in those with anxiety/depression in previous studies by our research group have shown amongst the best reliability in the data we report here: including the avoidance bias parameter (Mkrtchian et al., 2017) and the drift rate (Aylward et al., 2020). Even if parameters are completely stable over time, estimates of test-retest reliability are also bounded by how recoverable parameters are – many computational modelling papers validate models by testing the accuracy with which known parameter values can be recovered from data simulated using them. This can vary dramatically between models, and may also depend on task design (Pike & Robinson, 2022). We recommend that researchers using repeated measures perform parameter recovery simulations prior to data collection, in order to optimize test-retest reliability.

Importantly, the EZDDM model parameters seemed to have greater test-retest reliability over time than the 4-parameter drift diffusion model parameters did. This should serve as a caution to other researchers – the 4-parameter drift diffusion model provides an extra parameter, but this seems to be at the cost of greater reliability, and also requires model fitting. There are versions of the DDM with even more parameters (Ratcliff, 1978), which could be investigated in future work, but given that a simplified 4-parameter version does not show additional benefits over the EZDDM it is unlikely to be of significant value in improving reliability.

Embedding the covariance matrix (Haines et al., 2020) within the estimation procedure increased our estimates of the correlations of most of these parameters, as it uses the whole posterior distribution of parameters. However, this result is only useful if this embedding procedure is used in the analysis pipeline of randomized studies – if we only have access to the point estimate of the parameters for each individual, any effect found is limited by their standard test-retest reliability (Haines et al., 2020; Travers, 2022). Returning to the clinical trial example, it is possible that by embedding both the covariance matrix and a variable that represents the effect of the intervention, greater sensitivity to the effect can be obtained, by specifically accounting for the imprecision of the estimates and also specifying that individuals are likely to produce similar behaviour over time (Haines et al., 2020). However, embedding the parameters does not improve correlation estimates in all cases – in particular, approach bias shows a reduced correlation, as do the drift rate and starting bias in the drift diffusion model – but the confidence intervals for the embedded correlation and non-embedded correlation overlap with each other in all of these instances. Speculatively, the large confidence interval around the approach bias could be diagnostic – it seems plausible that many different values of the correlations of this parameter have similar posterior probability, so perhaps this parameter is superfluous.

We have multiple recommendations for other researchers on the basis of these results. Firstly, the ambiguous midpoint task is preferable to the go-nogo task if one has to be chosen to use as a measure of negative affective bias in a clinical setting – it produces fewer separate outcome measures (whether summary statistics or computational parameters are required), and all of these are generally more reliable than the worst-performing measures from the go-nogo task. We also suggest summary statistics in negative affective bias tasks are not substantially worse as reliable measures of negative affective biases than computational parameters estimated in the standard way, so researchers without computational modelling expertise do not necessarily need to acquire it. However, if teams do have this expertise, we recommend that the covariance matrix should be embedded, as this may improve reliability and allow the effect of the intervention to be estimated within-model, thus accounting for different sources of variance.

### Limitations

We used only two illustrative affective bias tasks in this study, chosen as they are well-characterised computationally, so our findings might not generalize to all affective bias measures. These two tasks differ in several important aspects: the ambiguous midpoint task involves training, but the go-nogo task does not, which may mean that there is a greater impact of practice on performance at time 2 and thus lower estimates of reliability. Equally, the probabilistic structure of the outcomes are different: in the ambiguous midpoint task, the ambiguous circle is rewarded at 50%, but the large and small circles are fully deterministically rewarded. On the other hand, all stimuli in the go-nogo task are probabilistically reinforced. Additionally, emotional faces are presented as feedback in the go-nogo task, which may cause specific difficulties in responding in clinical populations, especially those where social feedback is a trigger for symptoms (e.g. social anxiety disorder). However, it is worth noting that the purpose of this paper is not to directly compare the two tasks, but to investigate whether using computational parameters improves the reliability of affective bias measures.

However, previous research in a developmental sample has indicated low test-retest reliability of a similar go-nogo task to the one we use here (Moutoussis et al., 2018). In particular, they found that only the go bias parameter correlation over time was significantly greater than 0, with a similar n of 61 in their ‘short-term follow up’ sample. In contrast, both of our sensitivity parameters, avoidance bias and go bias showed significant correlations in our model (though note that our winning model was not considered in their model-fitting procedure).

Whilst our sample size was justified prior to data collection (see Supplementary material), our estimates of reliability are more imprecise than might be desirable – as can be seen by the width of the confidence intervals. Relatedly, many randomized controlled trials include a greater number of participants per cell than the n of 58 we report here (Jakubovski et al., 2019). Finally, within hierarchical computational models, the extent to which parameter estimates display ‘shrinkage’ towards the group-level prior depends on the sample size: estimates from larger samples will be estimated with less reliance on the group-level hyperparameters, which may improve how reliably parameters are estimated, and increase test-retest reliability.

Relatedly, parameter estimation method and the overall parameterization of the model may artificially reduce reliability. Several points are of note here. The EZDDM model has an exact closed-form solution, so does not require estimation – therefore any method used should in theory return the same result. For the other models reported here, we used cutting-edge estimation (Markov-Chain Monte-Carlo) and a hierarchical estimation approach (Ahn et al., 2017). We also used model comparison to identify the best model from a family of reinforcement learning models in the go-nogo task (though, notably, this model differed to the best-fitting model found in previous work; Mkrtchian et al., 2017). However, it is of course possible that other parameterizations or estimation methods might improve test-retest reliability – and indeed, work to achieve this is ongoing (Zorowitz et al., 2023). This is particularly the case when considering model comparison – a model may be the best fit to the data for a single session, but this does not necessarily mean it is a reflection of real cognitive processes that persist over time, or that new, bespoke models might not provide improvements in the future.

Additionally, we excluded participants who seemed to be inattentive in their responses, based on either no responses in either task or using a single response key throughout. Participants who are inattentive might perform more randomly, thus increasing the variance of measures and perhaps resulting in inflated estimates of reliability in this study (although, conversely, if a participant performed perfectly randomly in both sessions their accuracy should be precisely 50% in any two-choice tasks). Excluding participants who seem inattentive might also have unforeseen effects and result in collider bias, particularly as inattention is one of the diagnostic criteria for many anxiety and mood disorders – removing those who seem inattentive might result in removing those with more severe anxiety and depression symptoms, thus potentially inducing effects where there are none, and removing others that should exist.

Furthermore, we only obtained mental health measures at time 1. It would have been useful to collect these measures at time 2, to understand whether levels of anxiety and depression remained stable over sessions, and also to identify participants who may have begun medication or treatment for mental illness in between. If negative affective bias varies with treatment, we might expect some variation with symptom levels to occur – which we could have accounted for in more sophisticated or subgroup analysis.

Finally, we performed this study online, which may have led to noisier participant behaviour than might be expected in a randomized controlled study of an intervention, where participants are monitored whilst performing tasks. However, online testing has corresponding benefits: all participants receive the exact same instructions, and thus their performance should be more uniform; and a greater sample size can be recruited more rapidly and with greater control over the interval between sessions than is possible in person. Relatedly, due to our use of Prolific to perform this study online, we reimbursed participants at a fixed rate based on our estimation of how long the task took to complete. Participants also gained no ‘bonus’ points or rewards based on performance. These two factors may have incentivised rapid and careless responding. However, it would be unjust to reimburse participants more if they performed well, given that the tasks we asked participants to perform are those on which those with worse mental health tend to be (on some measures) less accurate and less optimistic.

## Conclusion

In conclusion, performance on affective bias tasks has low to moderate test-retest reliability. Summary statistics showed generally moderate test-retest reliability, but computational modelling of these tasks did not generally improve reliability, and in some cases reduced it. There are some exceptions to both of these general statements, which may be informative in future attempts to improve the reliability of affective bias measurement.

## Additional File

The additional file for this article can be found as follows:

10.5334/cpsy.92.s1 Supplementary Material. Supplementary methods and results for this paper, including sample size justification, demographic variables, and model fitting details.
