# Supplementary material for: Test-Retest Reliability of Two Computationally-Characterised Affective Bias Tasks
Source: Comput Psychiatr. 2024 Dec 18;8(1):217–32. doi: 10.5334/cpsy.92 (PMC11661199; doi:10.5334/cpsy.92)
Supplement: Supplementary Material. — Supplementary methods and results for this paper, including sample size justification, demographic variables, and model fitting details. [file cpsy-8-1-92-s1.pdf]

Supplementary Material
Test-retest reliability of two computationally-characterised affective bias tasks

Pike, Alexandra C.[1]; Tan, Katrina[1]; Ansari, Hoda[1]; Wing, Michelle[1]; Robinson, Oliver J.[1,2*]

SAMPLE SIZE JUSTIFICATION

We performed an a-priori power analysis using G*Power (version 3.1.9.6), and determined that we would require a sample size of 50 as the number required to achieve 90% power (given an alpha of 0.05) to observe a minimally-interesting effect size of ρ=0.4 (corresponding to a fair or above test-retest reliability, using a one-sided test as we did not anticipate negative correlations), using 'Correlation: bivariate normal model' in the 'Exact' category of tests (Faul et al., 2009). Assuming attrition between times 1 and 2 and exclusions for inattention, we recruited a sample of 82 participants at time 1. 69 of these returned to complete session 2.

DEMOGRAPHIC AND MENTAL HEALTH QUESTIONS

Prior to completion of other questionnaires, we asked participants to complete a short questionnaire on demographic and mental health variables (note: this was a questionnaire they completed at the start of the study, rather than the Prolific screening questions, to ensure responses were up to date). Participants were not excluded on the basis of any of these responses. They were asked the country they were resident in, their age in years, and how many years of education they had completed. We also asked them which gender they identified as. Related to mental health, we asked whether they had ever been diagnosed with a mental or psychiatric illness, whether they'd ever specifically been diagnosed with anxiety or depression, and whether they had currently or previously taken medication for mental illness in general and anxiety or depression specifically.

From the data Prolific provides, we also collected 'Prolific scores' – these are out of 100, and are a metric of participant 'quality' – they are the number of studies that a participant has completed that have been 'approved', over the total number that have been either approved or rejected. Relevant descriptive statistics for these variables are shown below.

**Variable**	**Category**	**Count**
Gender	Female	34
	Male	23
	Nonbinary	1
Diagnosis of any mental illness	Yes	8
	No	58
Diagnosis of anxiety or depression	Yes	20
	No	38
Medication for any mental illness	Yes, currently	4
	Yes, in the past	7
	No	47
Medication for anxiety or	Yes, currently	5

depression		
	Yes, in the past	13
	No	40
Country of residence	UK/Great Britain/England/Scotland*	19
	Belgium	2
	Estonia	3
	Finland	1
	Germany	1
	Greece	3
	Hungary	4
	Ireland	1
	Italy	4
	The Netherlands	2
	Portugal	8
	Poland	6
	USA	3
	South Korea/UK	1
**Variable**	**Statistic**	Value
Age	Mean	28.0
	Standard deviation	8.43
Years of education	Mean	15.0
	Standard deviation	3.69
Prolific score	Mean	99.43
	Standard deviation	1.67
Interval between sessions	Mean	15.67
	Standard deviation	3.60

Supplementary Table 1: Table displaying descriptive statistics for demographic and mental health variables that were collected for this sample. *Note that participants submitted free-text responses, so responses of UK/Great Britain/England and Scotland were grouped together. Similarly, differences in capitalisation or naming (e.g. Netherlands vs. the Netherlands) were also grouped together. Individual-Level Performance

Supplementary Figure 1 shows individuals' summary statistic performances in all conditions of the two tasks, separated into time 1 and time 2.

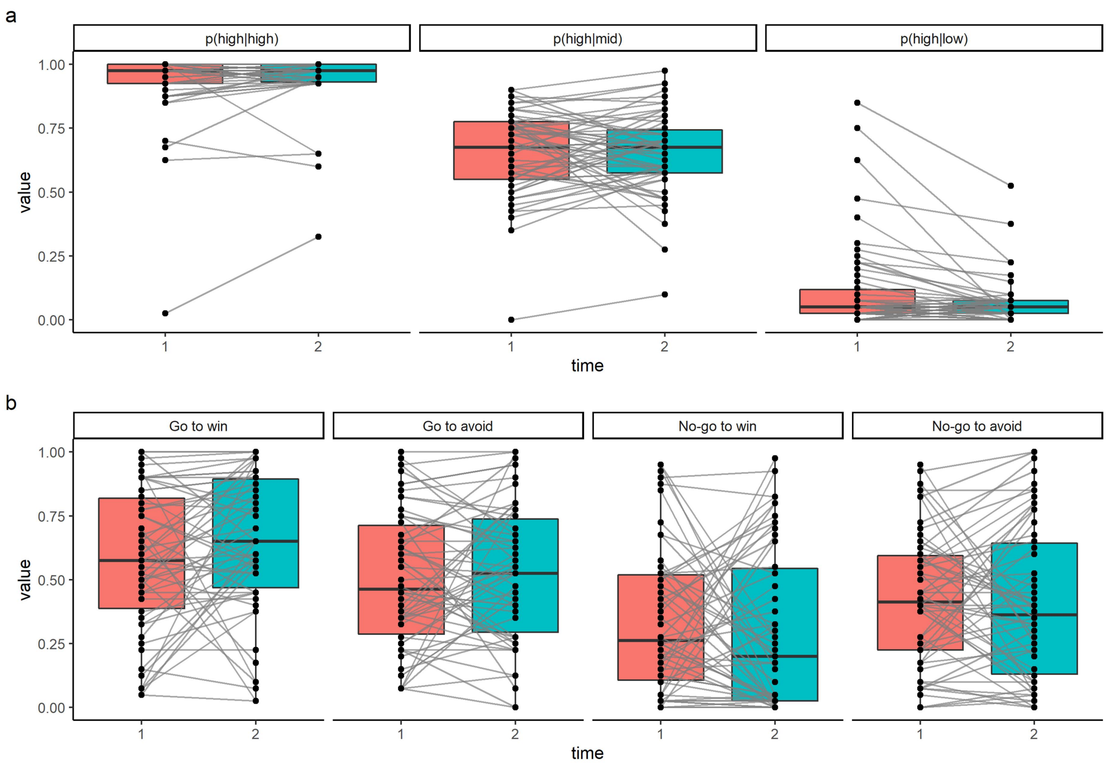

Supplementary Figure 1: Boxplots overlaid with individual data points for performance in all conditions of the two tasks. The middle line in the boxplot represents the median, the bottom and top lines represent the first (25th) and third (75th) quartiles. Each individual data point is shown by a black dot – grey lines join individuals' performance between time 1 and time 2.

SPLIT-HALF RELIABILITY

To assess the internal consistency of the tasks, we report their split-half reliability per session and per outcome measure. Split-half reliability was calculated by randomly sampling half of the trials for each participant (the same half was used for each participant) and calculating the key summary statistics over this subset of trials. We then performed a Pearson's correlation between these summary statistics for the two splits.

**Session**	**Task**	**Measure**	**Pearson's correlation**	
			**coefficient**	**p-value**
1	Go-nogo	Go to win accuracy	0.898	<0.0001
		Go to avoid accuracy	0.886	<0.0001
		Nogo to win accuracy	0.925	<0.0001
		Nogo to avoid accuracy	0.913	<0.0001
	Ambiguous midpoint	P(high|mid)	0.741	<0.0001
2	Go-nogo	Go to win accuracy	0.916	<0.0001
		Go to avoid accuracy	0.918	<0.0001
		Nogo to win accuracy	0.958	<0.0001
		Nogo to avoid accuracy	0.951	<0.0001
	Ambiguous midpoint	P(high|mid)	0.592	<0.0001

Supplementary Table 2: Split-half reliability displayed as Pearson's correlation coefficients for summary statistics estimated from a randomly selected 50% of trials for each affective bias task. Values greater than 0.4 are often described as moderate-good, less than 0.4 are poor. All of these values meet this threshold.

MODEL FITTING

Models were fit using hBayesDM (v1.1.1, all standard models) or cmdstanr (v 0.4.0, models in which the covariance matrix was embedded) in R (v4.0.2). Four chains were used for each model, with 10,000 iterations each.

Go-Nogo Task Models

For the go-nogo task, we formally compared the fit of seven different models. Four of these are derived from the hBayesDM package (Ahn et al., 2017), and three are adaptations of these models, based on previous work (Mkrtchian et al., 2017) (Supplementary Table 3).

Model name	Learning rate	Noise parameter	Sensitivity	Go bias	Pavlovian bias
gng_m1	1	1	1		
gng_m2	1	1	1	1	
gng_m3	1	1	1	1	
gng_m4	1	1	2 (reward and punishment)	1	1
gng_m5	1	1	2 (reward and punishment)	1	2 (approach and avoid)
gng_m6	2 (reward and punishment)	1	2 (reward and punishment)	1	2 (approach and avoid)
gng_m7	2 (reward and punishment)	1	2 (reward and punishment)	1	1

Supplementary Table 3: Model specifications for go-nogo models. Their name, the number of learning rates, noise parameters, sensitivity parameters and different biases are described.

The best-fitting model (see below, gng_m5) can be expressed using the following equations:

$$Q_{t+1(a_t,s_t)} = Q_{t(a_t,s_t)} + LearningRate * (sensitivity * outcome_t - Q_{t(a_t,s_t)})$$

$$V_{t+1(s_t)} = V_{t(s_t)} + \alpha * (sensitivity * outcome_t - V_{t(s_t)})$$

where $sensitivity = RewardSensitivity\ if\ outcome \geq 0; PunishmentSensitivity\ otherwise$

Subsequently, these learnt values were converted into action weights:

$$weight_{t(a_t)} = Q_{t(a_t,s_t)} + ActionBias + PavlovianBias * V_{t(s_t)}$$

where $PavlovianBias = ApproachBias\ if\ V_{t(s_t)} \geq 0; AvoidBias\ otherwise$

where $ActionBias\ and\ PavlovianBias\ were\ set\ to\ 0\ if\ the\ action\ was\ no-go$

These weights were then converted into a probability of taking a specific action:

$$P_{t(a_t)} = (1 - noise) * \frac{exp(weight_{t(a_t)})}{\sum_{a=1}^{n} exp\,(weight_{t(a_t)})} + \frac{noise}{n}$$

Note that Q refers to the Q-value of a given state s (i.e. stimulus) and action a (go or no-go) combination, V refers to the value of a particular state regardless of action, t indicates a specific

trial, *P* indicates the probability of a response, and *outcome* is the points received by the participant on that trial (could be 1, 0 or -1).

The sampling statement within the Stan code incremented the target log probability density using the Bernoulli distribution (Bernoulli log probability mass function) for the probability of a 'go' response (given the cue, participant and trial). Note that these sampling statements in Stan drop constant additive terms.

This model used non-centred parameterisations. All error terms were drawn from the standard normal distribution. Both learning rate and noise parameters had group-level means drawn from *Normal(0,1)* distributions, and group-level standard deviation drawn from *Normal(0,0.2)* distribution. They were then transformed to be within the interval [0,1] using the Phi_approx function in Stan. All go bias terms (approach bias, avoid bias, and go bias) had group-level means drawn from the *Normal(0,10)* distribution, and standard deviations drawn from the *Cauchy(0,1)* distribution. Reward and punishment sensitivity had their group-level means drawn from *Normal(0,1)* distributions, and group-level standard deviations drawn from the *Normal(0,0.2)* distribution, and were then transformed to being on the positive infinite number line using an exponential transformation.

Ambiguous Midpoint Task Models

As noted in the main manuscript, we fit both an approximation to the drift-diffusion model (Wagenmakers et al., 2007), referred to as the 'EZ-DDM', and a 4-parameter drift-diffusion model instantiated in hBayesDM (Ahn et al., 2017).

EZ-DDM

The model equations for the EZ-DDM are described in detail in Wagenmakers et al. 2007, but the simple forward model is summarized here (note that this has an analytic solution, so does not require model fitting):

$$y = \exp(-BoundarySeparation * DriftRate)$$

$$Accuracy = \frac{1}{y+1}$$

$$RTMean = NonDecisionTime + \left(\frac{BoundarySeparation}{2 * DriftRate}\right) * \left(\frac{y-1}{y+1}\right)$$

$$RTVariance = \left(\frac{BoundarySeparation}{2 * DriftRate^3}\right) * \left(\frac{1 - (2 * BoundarySeparation * DriftRate * y) - y^2}{(y+1)^2}\right)$$

4-parameter DDM

The 4-parameter-DDM equation can be written as follows:

$$ResponseTimeUpper = Wiener(BoundarySeparation, NonDecisionTime, StartingBias, DriftRate)$$

Where Wiener indicates Wiener First Passage Time Distribution. More fully, the probability density function is:

$$Wiener(ResponseTimeUpper \mid BoundarySeparation, NonDecisionTime, StartingBias, DriftRate)$$
$$= \frac{BoundarySeparation^3}{(ResponseTimeUpper - NonDecisionTime)^{\frac{3}{2}}} *$$
$$\exp\left(-DriftRate * BoundarySeparation * StartingBias - \frac{DriftRate^2 * (ResponseTimeUpper - NonDecisionTime)}{2}\right)$$
$$* \sum_{k=-\infty}^{\infty} (2k + StartingBias) * \varphi\left(\frac{2k * BoundarySeparation + StartingBias}{\sqrt{ResponseTimeUpper - NonDecisionTime}}\right)$$

Where $\varphi(x)$ denotes the standard normal density function.

This model also used non-centred parameterization. Note that all group-level means and error terms are drawn from a *Normal(0,1)* distribution, and all standard deviations are drawn from a *Normal(0,0.2)* distribution. Starting Bias was transformed using Phi_approx to be within the [0,1] interval, Boundary Separation was transformed to be on the positive infinite number line using an exponential transform, Drift Rate was not transformed, and Non-Decision Time was transformed to be within the [0,1] interval using Phi_approx, and then multiplied by the lower bound of RT (set by the authors to 0.1 seconds, the default) multiplied by the difference between the individual's smallest RT and the lower bound of RT.

Model comparison

Models for the Go-Nogo task were compared using the integrated BIC (Huys et al., 2011) using a conservative estimate of the number of parameters per individual that does not account for partial pooling. The best fitting model is considered to be the one with the lowest integrated BIC total.

The best fitting model at time 1 was gng_m5, and at time 2 was the same (Supplementary Figure 2).

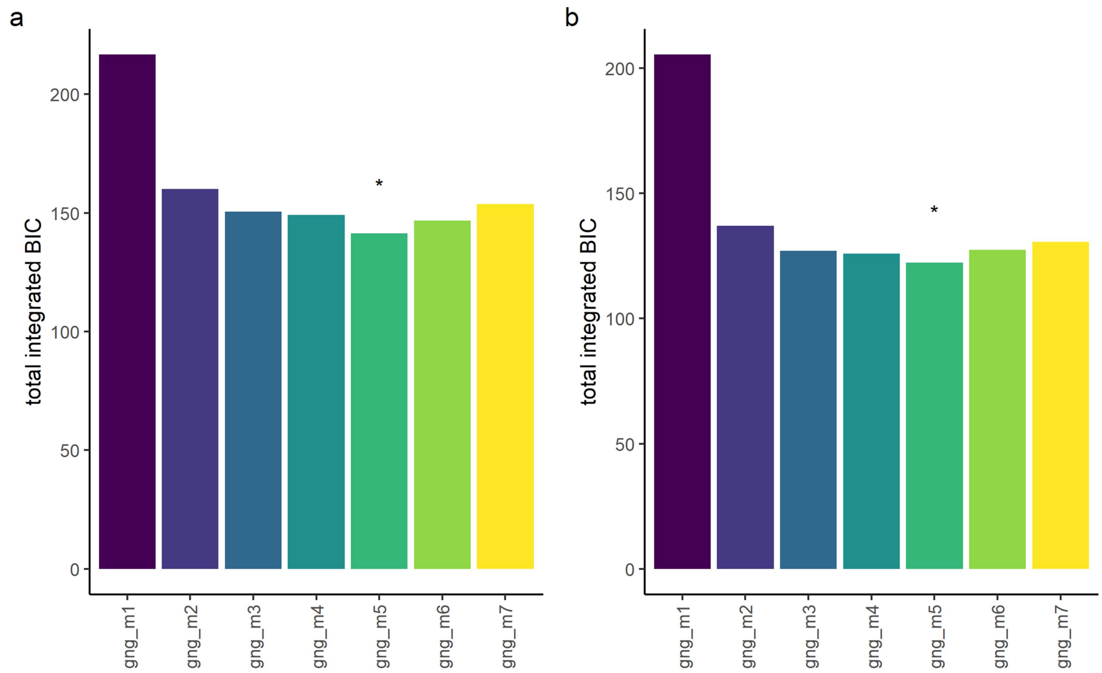

Supplementary Figure 2: Total integrated BIC scores at time 1 (a) and time 2 (b) for the go-nogo models fit to participant choice data. The lowest integrated BIC score at each time is denoted with an asterisk.

Individual-level parameters

Supplementary Figure 3 shows the individual parameter estimates for individuals, split by times 1 and 2 and per parameter, for each of the affective bias tasks. Whilst there is some indication (particularly for the model fit to the Go-Nogo task) that within-session variance may differ between sessions 1 and 2, these differences are not systematic or in the same direction, thus indicating that computing ICCs may still be informative.

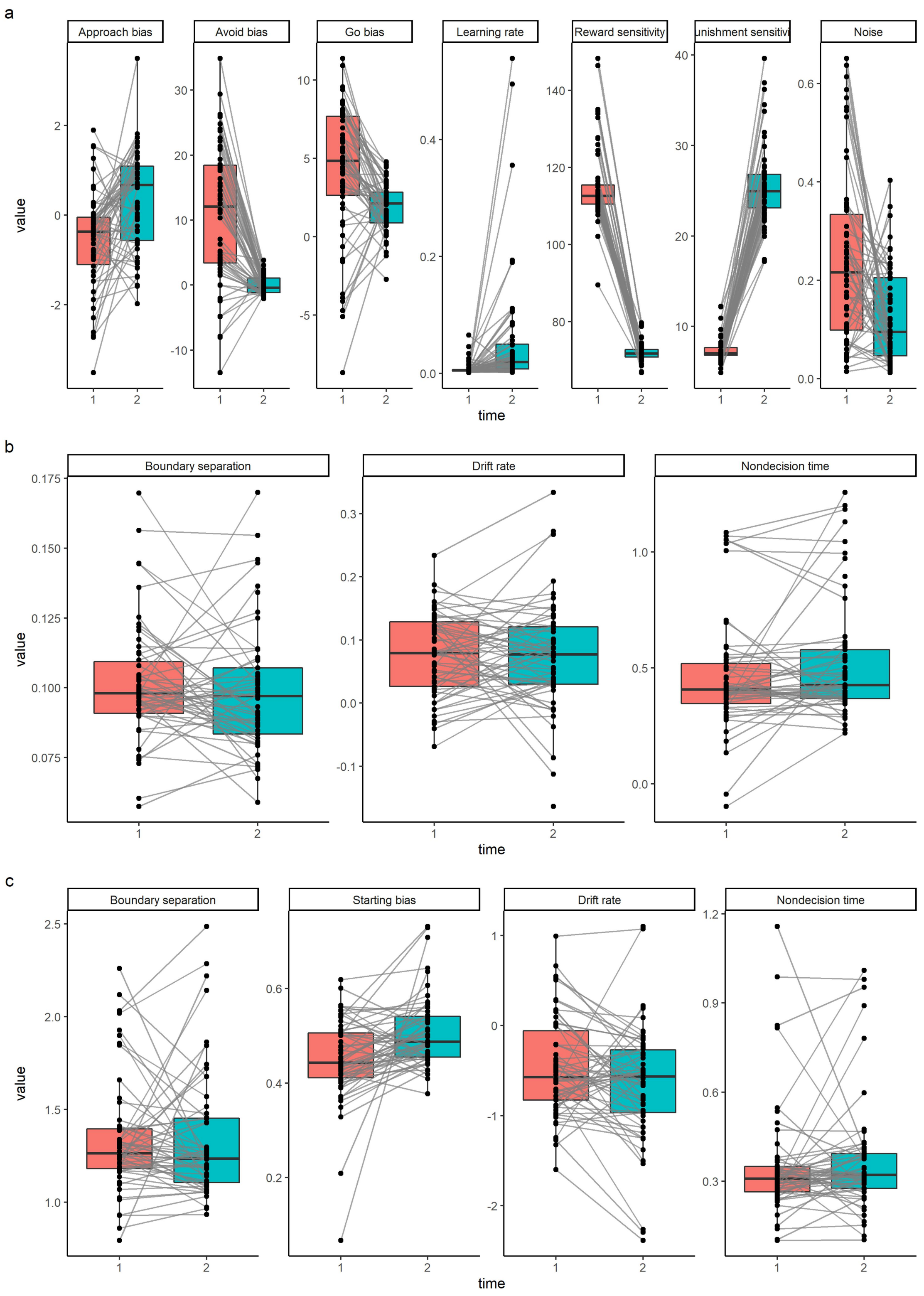

Supplementary Figure 3: Boxplots overlaid with individual data points for each computational parameter. A) The winning model for the Go-Nogo task, B) the EZ-DDM for the Ambiguous Midpoint task, C) the 4-parameter-DDM fit to the ambiguous midpoint task. The middle line in the boxplot represents the median, the bottom and top lines represent the first (25th) and third (75th) quartiles. Each individual data point is shown by a black dot – grey lines join individuals' parameter estimates between time 1 and time 2.

Embedding correlation matrix

To embed the correlation matrix within the generative modelling framework, we took the same approach as in previous work (Haines et al., 2020). This involves including a separate Cholesky factor version of the group-level correlation matrix for each parameter at time 1 and time 2, and specifying group level parameter means and standard deviations as would be typical in a non-centred hierarchical parameterisation, alongside individual level parameter errors. Within the 'transformed parameters' block in the Stan model code, individual offsets are calculated using diag_pre_multiply to multiply the Cholesky factor and the standard deviation for each parameter, and then multiplying this by the individual error terms. Subsequently, the group-level means are added to the individual offsets and transformed if necessary to ensure the parameters are appropriately bounded. The priors over the Cholesky factors were the LKJ correlation matrix, and all other priors were the standard normal distribution. To obtain the correlation matrix, the Cholesky factors are multiplied by their transpose in the generated quantities block. Rather than using a single multivariate normal distribution across all parameters, we used separate normal distributions and matrices for each, due to a known issue where correlations can be pooled towards 0 as more parameters are added (see https://discourse.mc-stan.org/t/pairwise-alternative-to-multivariate-normal-isnt-behaving-for-the-hierarchical-case-help/22792). The code used to implement this can be found with our open data.

SUPPLEMENTARY REFERENCES

Ahn, W.-Y., Haines, N., & Zhang, L. (2017). Revealing Neurocomputational Mechanisms of Reinforcement Learning and Decision-Making With the hBayesDM Package. *Computational Psychiatry*, *1*, 24–57. https://doi.org/10.1162/CPSY_a_00002

Faul, F., Erdfelder, E., Buchner, A., & Lang, A.-G. (2009). Statistical power analyses using G*Power 3.1: Tests for correlation and regression analyses. *Behavior Research Methods*, *41*(4), 1149–1160. https://doi.org/10.3758/BRM.41.4.1149

Haines, N., Kvam, P. D., Irving, L. H., Smith, C., Beauchaine, T. P., Pitt, M. A., Ahn, W.-Y., & Turner, B. (2020). *Learning from the Reliability Paradox: How Theoretically Informed Generative Models Can Advance the Social, Behavioral, and Brain Sciences*. PsyArXiv. https://doi.org/10.31234/osf.io/xr7y3

Huys, Q. J. M., Cools, R., Gölzer, M., Friedel, E., Heinz, A., Dolan, R. J., & Dayan, P. (2011). Disentangling the Roles of Approach, Activation and Valence in Instrumental and Pavlovian Responding. *PLoS Computational Biology*, *7*(4), e1002028. https://doi.org/10.1371/journal.pcbi.1002028

Mkrtchian, A., Aylward, J., Dayan, P., Roiser, J. P., & Robinson, O. J. (2017). Modeling Avoidance in Mood and Anxiety Disorders Using Reinforcement Learning. *Biological Psychiatry*, *82*(7), 532–539. https://doi.org/10.1016/j.biopsych.2017.01.017

Wagenmakers, E.-J., Van Der Maas, H. L. J., & Grasman, R. P. P. P. (2007). An EZ-diffusion model for response time and accuracy. *Psychonomic Bulletin & Review*, *14*(1), 3–22. https://doi.org/10.3758/BF03194023
